# The predictive role of hope and social relational quality in disability acceptance among Iranian patients under hemodialysis

**DOI:** 10.1186/s12882-023-03161-x

**Published:** 2023-04-20

**Authors:** Nilofar Pasyar, Mostafa Jowkar, Masoume Rambod

**Affiliations:** 1 Community Based Psychiatric Care Research Center, Nursing and Midwifery School, Shiraz University of Medical Sciences, Shiraz, Iran; 2 Student Research Committee of Shiraz University of Medical Sciences, Shiraz, Iran

**Keywords:** Disability acceptance, Communication, Hemodialysis, Hope

## Abstract

**Background:**

Patients undergoing hemodialysis face disabilities that its acceptance may influenced by several factors. This study aimed to determine the predictive role of hope and quality of social relationship on accepting disability amongst patients undergoing hemodialysis.

**Methods:**

This cross-sectional study was conducted on 120 hemodialysis patients referred to hemodialysis centers in Nemazi and Shahid Faghihi hospitals and Imam Reza Clinic in Shiraz. Snyder Hope Scale, Acceptance of Disability Scale (ADS), and Social Relational Quality Scale (SRQS) were used for data collection. The data were analyzed through the Smart PLS-3 and SPSS software using Pearson’s correlation and multiple linear regression analysis tests, and confirmatory factor analysis.

**Results:**

Face, content, and construct validities and internal consistency of the Persian version of ADS and SRQS were confirmed. The patients’ mean score of hope was 38.83 (SD = 4.35), which was not desirable. Their mean score of SRQS was 45.45 (SD = 3.87), which was at the moderate level. Nonetheless, the mean score of disability acceptance (66.01 (SD = 7.15)) was lower than expected. The results showed disability acceptance was associated with having good level of hope (β = 0.44, p = 0.002) and social relationship (β = 0.31, p = 0.04).

**Conclusions:**

Hope and social relational quality predicted the acceptance of disabilities. Therefore, designing interventions to promote hope and social relationship in hemodialysis patients may increase their disability acceptance.

## Introduction

End Stage Renal Disease (ESRD) is a main public health problem worldwide [1]. Its prevalence has been reported as 242 per million worldwide, with 8% being added annually [2]. In Iran, nearly 1200–1600 people are diagnosed with ESRD every year [3]. ESRD and its treatments such as hemodialysis lead to annoying complications [4] including intra-dialytic hypotension, muscle cramps, dialysis disequilibrium syndrome, dialyzer reaction, hemolysis, and air embolism. It also leads to other nonspecific complications such as nausea and vomiting, headache, chest and back pain, and itching [5]. These physical complications are a large burden of symptoms in ESRD patients [6]. Moreover, patients usually experience a reduced quality of life due to the long-term course of hemodialysis treatment and its complications. This disease can cause psychological changes, stress [7], anxiety, and depression [8]. It reduces the leisure time and quality of relationships with family and friends [9].

## Background

Based on Snyder’s hope theory, hope as a cognitive and affective element may be effective in helping patients to deal with these complications. According to Snyder’s hope theory, to be hopeful, people need to focus on their thoughts, develop strategies to achieve goals, and be motivated to meet these goals [10]. Hope may enable people to improve their physical and mental health and live their life more efficiently [11]. Hopeful people can take care of themselves more desirably [12].

One of the challenges facing ESRD patients is the disruption of interpersonal relationships [7]. A study on hemodialysis patients showed that non-family networks, such as friends, decrease over time [13]. Having a larger social network was associated with higher participation-seeking preference and lower levels of anxiety [13]. In addition, closer and more satisfying relationships or high-quality social relationships were correlated to better psychological well-being [13].

Disability is another challenge facing ESRD patients undergoing hemodialysis. It refers to “a person, some of whose normal daily activities are impeded or hindered due to the alteration of their intellectual or physical functions” [15]. It occurred in ESRD patients because of comorbid conditions and complications, which impaired their daily activities [16]. It was reported that 20.5% and 14.0% of ESRD patients on hemodialysis lived about 2.0 and 1.3 years with moderate and severe disabilities, respectively [14]. As the disability in ESRD patients undergoing hemodialysis increases, their physical and mental well-being decreased [17]. Moreover, non-disabled and mildly disabled ESRD patients undergoing hemodialysis had a higher survival rate compared to those with moderate or severe disabilities [17]. Moreover, disability was associated with more cardiovascular events in hemodialysis patients [18]. Acceptance of disability is a key factor in adapting to the course of the disease and these complications. Wright (1983) described disability acceptance as “a series of changes in values, in which an individual extends his or her range of values, places less importance on critical thinking about physical abilities, and places increased importance on one’s remaining abilities” [19].

Patients with acceptance realize that they can successfully overcome their life restrictions [20]. Chiang et al. revealed that patients with chronic kidney disease with lower disability acceptance scores were more exposed to advanced stages of the disease such as dialysis and death. Hence, they suggested these patients be screened for disabilities [21]. Hope has been often reported as a significant state of mind, influencing the patients’ performance and outlook on life. Such a concept can effectively improve the patients’ acceptance during dialysis treatment, regular follow-up and clinical examinations. Furthermore, patients’ problems and disabilities raise the importance of the quality of social communication in their lives. Particularly, patients’ appropriate communication and interpersonal skills with medical staff may reduce their therapeutic burden and enable them to self-manage the disease. It may also facilitate their acceptance of the disabilities [22–25]. According to Zhang et al., there is a positive association between social relational quality and disability acceptance. In other words, if the disease is under control and patients can have an appropriate assessment of their physical appearance and emotions, they will be able to get along well with their family and friends [26].

Up to now, few studies have been conducted on disability acceptance amongst patients with chronic diseases [22–25] and chronic kidney disease [21]. In addition, a limited number of studies have examined the relationships among hope, social relational quality, and acceptance of disability in ESRD patients undergoing hemodialysis. Moreover, some studies have been carried out in other parts of the world except the Middle East countries such as China, Japan, and Western countries. Moreover, based on the researchers’ expertise in the field of ESRD and hemodialysis, the number of these patients has increased significantly in the last two decades in Iran, one of the Middle East countries. There is a large number of ESRD patients undergoing hemodialysis in Iran. Moreover, there were various challenges, lack of knowledge regarding hope, social relational quality, and acceptance of disability and their relationships in ESRD patients undergoing hemodialysis. In addition, the promotion of evidence-based practice provided the basis for answering the question of whether hope and social relational quality can predict disability acceptance in Iranian patients undergoing hemodialysis.

The hypotheses posed were as follows:

1. Hope, social relational quality, and acceptance of disability are associated with demographic and clinical characteristics.
2. Hope and social relational quality are associated with disability acceptance in Iranian patients undergoing hemodialysis.
3. Hope and social relational quality will predict disability acceptance in Iranian patients undergoing hemodialysis.

## Methodology

### Study design and setting

The present cross-sectional study was conducted in the hemodialysis centers of Nemazi Hospital, Shahid Dr. Faghihi Hospital, and Imam Reza Boarding Clinic in Shiraz in 2019.

### Participants

The study participants included 120 patients on hemodialysis selected via systematic sampling. The inclusion criteria of the study were being over 18 years, having been under hemodialysis treatment for at least six months, and undergoing hemodialysis at least twice and at most three times per week. Patients were excluded if they had experienced emotional crises such as the death of their loved ones and divorce in the past six months.

### Study size

Based on the research entitled “Correlation between acceptance of disability and social relational quality” by Zhang et al. [26], r = 0.32, α = 0.05, and 1-β = 0.90, the minimum sample size was estimated as 98 patients. Considering the drop-out rate of 20%, we increased it to 118 ≅ 120.

### Data sources/measurements

The data were collected using a demographic and clinical characteristics form, Snyder Hope Scale, Acceptance of Disability Scale (ADS), and Social Relational Quality Scale (SRQS). The demographic and clinical characteristics form included information such as age, gender, level of education, marital status, employment status, duration of dialysis, number of dialysis sessions per week, duration of dialysis per day, ability to perform daily tasks, and use of assistive devices.

Snyder Hope Scale is a 12-item test designed by Snyder et al. (1991) and used to measure hope as a relatively constant personality trait. This tool is divided into two subscales of agency and pathways thinking. “Items 2, 9, 10, and 12 make up the agency subscale, and items 1, 4, 6, and 8 make up the pathway subscale.” The participants were required to rate their agreement or disagreement with each statement on an eight-point Likert scale ranging from definitely true (score = 8) to definitely false (score = 1). Thus, the total score of the scale could range from 8 to 64. Scores of 40–48 show hopeful, 48–56 indicate moderately hopeful, and 56 or higher are high hope states [27]. The internal consistency of the scale was 0.84, ​​and its test-retest reliability was 0.80 in eight weeks. Additionally, the internal consistency was 71–76% for the agency thinking subscale and 63–80% for the pathways subscale [28]. Pasyar et al. (2020) reported the reliability of the Persian version of this tool to be 0.885 [29]. Besides, Kermani et al. (2011) assessed its construct validity using factor analysis, showing that the scale had a two-factor structure including agency and pathways thinking [24]. The reliability of this scale was reported to be 0.80 in hemodialysis patients [30]. In the present study, Cronbach’s alpha reliability for the total scale, the agency thinking subscale, and the pathways subscales were 0.86, 0.80, and 0.82, respectively.

The SRQS was used to assess the patients’ relationships with their families and friends. It contains 17 questions scored using a four-point Likert scale ranging from strongly disagree (score 1) to strongly agree (score 4). Thus, the total score of this questionnaire ranges from 17 to 68. It includes three subscales of family intimacy, family commitment, and friendship, with Cronbach’s alpha coefficients of 0.80, 0.82, and 0.75, respectively [31]. In this study, after translating and reviewing the Persian questionnaire, we selected the most appropriate Persian translation that best suited the original form. Moreover, the psychometric properties of the Persian version of SRQS were evaluated.

The revised ADS was used. This scale was designed by Linkowski (1971) in English to measure the acceptance among patients having a physical disability. It was developed based on Wright’s (1983) theory to assess the feeling, values, and emotions associated with disability. It was designed based on “Dembo, Leviton, and Wright’s (1956) concept of acceptance of loss” [32]. ADS was used in physically disabled students [32], patients on dialysis [21], and those with stroke [33], chronic illness and disabilities [34], etc. As in previous studies, we used ADS in dialysis patients, as one of the questionnaires employed in this study. In the current study, an edited version of this scale compiled in 2004 by Groomes and Linkowski was used. It contains 32 questions scored using a four-point Likert ranging from strongly disagree (score = 1) to strongly agree (score = 4). Thus, the total score of the scale ranges from 32 to 128, with higher scores indicating greater disability acceptance [35]. The scores 32–64, 65–96, and 97–128 indicate low, medium, and high levels of disability acceptance, respectively [36]. This scale assesses four domains, namely “enlargement of scope of values”, “subordination of physique”, “containment of disability effects”, and “transformation from comparative to asset values”. These subscales indicate “Wright’s successful coping with disability paradigm”. Among the 32 items, 10 reflect positive meaning, and 22 items reflect negative meaning. Groomes and Linkowski approved the construct validity and internal consistency of the revised ADS [35]. In this study, after translating and reviewing the Persian version of ADS, the psychometric properties of the Persian version of ADS were evaluated.

In this study, for handling and preventing the missing data, the following planning was done; before completing the questionnaires, the research assistant explained how the patients should fill out the questionnaires carefully. Moreover, as the subjects filled out the questionnaires during the hemodialysis when they felt well, they had the right time and place to answer the questions.

### Ethical considerations

This study was carried out after obtaining approval from the Ethics Committee of Shiraz University of Medical Sciences (IR.SUMS.REC.1398.93) according to the Helsinki Declaration. Thus, at the beginning of the work, the participants were informed about the study objectives and their voluntary nature of participation in the study; also, their written consent forms were obtained. They were notified about their right to discontinue participation at any stage of the study as well. Coding and confidentiality of the participants’ information were admitted during the study. Therefore, sharing and usage of data were anonymous. No financial compensation was paid to the hemodialysis patients in the study. The subjects were appreciated for their involvement in this study.

### Statistical methods

To assess the psychometric properties of the Persian version of SRQS and ADS, we used Smart-PLS 3 to do confirmatory factor analysis (CFA). Data analyses were done through SPSS software, version 22 using descriptive statistics (percentage and frequency) and inferential statistical methods (Independent t-test, Analysis for Variance (ANOVA) test, Pearson’s correlation, and multiple linear regression analysis). Based on the researchers’ experience in hemodialysis wards, age and gender might be the confounders of disability acceptance. Therefore, they added these variables to the regression model. The normality of the data was assessed using Q-Q and P-P plots; the variables of this study had an approximately normal distribution. Therefore, to do multiple linear regression analysis, we considered the variables as normally distributed.

## Results

Psychometric properties of SRQS and ADS.

### Translation process

To use SRQS and ADS in the Persian language, we used two forward translations and one backward translation. This was conducted by three experts in the field of nursing, English language editing, and English language education. One of these experts was an Iranian who had lived in England for 10 years. Moreover, three researchers in this study collaborated in this process. After this step, five ESRD patients were interviewed to assess the quality of cognition and understanding of each item of the scales.

### Face and content validities

In the next step, the face and content validities were assessed. Face validity was conducted by 10 experts to evaluate the importance of each item (Impact score). As Tables 1 and 2 show, all the items in both scales obtained an impact score of more than 1.5 and remained for the next analyses. For content validity, the content validity rate (CVR), and item-content validity index (I-CVI) were determined. CVR > 0.60 and CVI ≥ 0.80 for each item were acceptable [37], and these items remained in the study. As Table 1, and 2 show, all the items in both scales had acceptable CVI and CVR, so all the items remained.

**Table 1:** Impact score, content validity rate (CVR), content validity index (CVI), average variance extracted (AVE), and composite reliability (CR) of Social Relational Quality Scale

	Item number	Impact score	CVI		CVR	AVE	AVE Each Domain	CR
Family intimacy	Q1	5	0.80		1	0.67	0.60	0.81
	Q2	5	1		1	0.74		
	Q3	4.9	0.80		1	0.62		
	Q4	5	1		1	0.66		
	Q5	4.9	1		1	0.64		
	Q6	5	1		1	0.54		
	Q7	5	1		1	0.52		
Family commitment	Q8	5	0.80		1	0.76	0.53	0.72
	Q9	5	0.80		1	0.71		
	Q10	5	1	1		0.50		
	Q11	5	1	1		0.72		
	Q12	4.9	0.80	1		0.72		
	Q13	5	1	1		0.50		
Friendships	Q14	5	1	1		0.75	0.72	0.87
	Q15	5	0.80	1		0.80		
	Q16	5	1	1		0.80		
	Q17	5	0.80	1		0.80		

**Table 2:** Impact score, content validity rate (CVR), content validity index (CVI), average variance extracted (AVE), and composite reliability (CR) of Acceptance of Disability Scale

	Item number	Impact score	CVI	CVR	AVE	AVE Each Domain	CR
Containment of disability effects	Q1	4.8	0.80	1	0.45	0.70	0.77
	Q11	4.8	0.80	1	0.50		
	Q15	4.8	1	1	0.53		
	Q17	4.8	1	1	0.53		
	Q20	4.9	1	1	0.54		
	Q22	4.9	0.80	1	0.60		
	Q27	4.9	0.80	1	0.58		
	Q30	4.8	1	1	0.69		
Transformation from comparative to asset values	Q2	4.9	0.80	1	0.62	0.68	0.72
	Q4	4.8	0.80	1	0.70		
	Q7	4.8	1	1	0.60		
	Q10	4.9	1	1	0.67		
	Q13	4.9	0.80	1	0.54		
	Q19	4.8	1	1	0.50		
	Q23	4.8	1	1	0.53		
	Q26	4.9	1	1	0.50		
	Q28	4.9	0.80	1	0.55		
Enlargement of the scope of values	Q3	4.8	1	1	0.46	0.62	0.81
	Q6	4.9	1	1	0.54		
	Q12	4.8	1	1	0.54		
	Q16	4.8	1	1	0.68		
	Q18	4.9	0.80	1	0.63		
	Q21	4.9	1	1	0.52		
	Q25	4.8	0.80	1	0.60		
	Q29	4.8	1	1	0.50		
	Q32	4.8	1	1	0.62		
Subordination of physique	Q5	4.8	1	1	0.69	0.73	0.74
	Q9	4.8	1	1	0.66		
	Q14	4.9	0.80	1	0.50		
	Q24	4.8	0.80	1	0.46		
	Q31	4.8	0.80	1	0.68		

### Confirmatory factor analysis (CFA)

Confirmatory factor analysis (CFA) using Smart-PLS 3 was used to evaluate the construct validity. As reported in Tables 1 and 2, average variances extracted (AVEs) of the items in both scales were higher than 0.45; therefore, the convergent validity of the construct of both scales was adequate.

### Internal consistency

As Table 1 indicates, all domains of SRQS had composite reliability (CR) ranged between 0.71 and 0.87, which were higher than 0.70 and Cronbach’s alpha of the scale was 0.77; therefore, its internal consistency was confirmed. Moreover, as Table 2 shows, all domains of ADS had CR ranged 0.72–0.82 which were higher than 0.70, and Cronbach’s alpha of the scale was 0.80; therefore, its internal consistency was confirmed.

#### Participants’ demographic and clinical characteristics

Most of the participants were male (69.2%), and their mean age was 54.05 years (SD = 11.89), with a range of 25–76 years. Additionally, most of the participants were married (84.2%) and 12.5% of them were employed (Table 3).

**Table 3:** The demographic and clinical characteristics of the hemodialysis patients

Variables		Number (%)
Gender	Male	83 (69.2)
	Female	37 (30.8)
Marital status	Single	13 (10.8)
	Married	101 (84.2)
	Divorced or widowed	6 (5)
Education level	Illiterate	24 (20)
	Primary and secondary schools	38 (37.1)
	High school and diploma	25 (20.8)
	Academic	33 (27.5)
Employment status	Unemployed	31 (25.8)
	Homemaker	31 (25.8)
	Retired	43 (35.8)
	Employed	15 (12.5)
Use of assistive devices	Nothing	87 (72.5)
	Canes and crutches	27 (21.15)
	Walkers	6 (5)
Ability to perform daily tasks	Excellent	13 (10.8)
	Good	61 (50.8)
	Fair	45 (37.5)
	Poor	1 (0.8)
Number of hemodialysis sessions per week	Two times	25 (20.8)
	Three times	95 (79.2)

#### Mean scores of hope, social relational quality, and disability acceptance

The patients in this sample were not hopeful as their mean score of hope was 38.83 (SD = 4.35), as the mean scores of the scale, were less than 40. Moreover, the mean score of social relational quality was 45.45 (SD = 3.87). Half of the expected score in this scale was 42.5. Therefore, the patients had a moderate social relational quality. The mean score of disability acceptance was 66.01 (SD = 7.15) in the patients undergoing hemodialysis, which was at the medium level. The mean scores of the subscales of the main study variables are shown in Table 4.

**Table 4:** The mean scores of hope, social relational quality, and acceptance of disability and their dimensions in the hemodialysis patients

Variables	Range	Mean (SD)
Hope	26–49	38.83 (4.35)
*Dimensions of hope*		
Agency thinking	12–26	19.59 (2.53)
Pathways thinking	12–24	19.24 (2.24)
Social relational quality	36–62	45.45 (3.87)
*Dimensions of social relational quality*		
Family intimacy	7–14	10.96 (0.99)
Family commitment	18–28	21.58 (1.77)
Friendships	10–20	12.9 (2.06)
Acceptance of disability	28–87	66.01 (7.15)
*Dimensions of acceptance of disability*		
Enlargement of the scope of values	12–24	15.87 (2.19)
Subordination of physique	10–23	16.79 (2.05)
Containment of disability effects	10–21	16.35 (2.08)
Transformation from comparative to asset values	12–23	17.27 (1.92)

#### The association between hope, social relational quality, and acceptance of disability and demographic and clinical characteristics

The results of the tests to examine the association between hope, social relational quality, and acceptance of disability and demographic and clinical characteristics revealed no significant association between the examined variables, expect using assistive devices, and education level. The mean score of disability acceptance was significantly higher in the people who did not use any aids to move compared to others (p < 0.001). Moreover, patients with academic degree reported highest mean score of hope (p = 0.04) (Table 5).

**Table 5:** The association between hope, social relational quality, and acceptance of disability and demographic and clinical characteristics

Demographic and clinical characteristics		Hope Mean (SD)	Social relational quality Mean (SD)	Acceptance of disability Mean (SD)
Gender	Male	38.66 (4.63)	45.5 (0.44)	65.44 (7.77)
	Female	39.21 (3.68)	45.29 (0.59)	67.29 (5.38)
	Test P	t = − 0.64^a^ P = 0.52	t = 30^a^ P = 0.76	t = − 1.31^a^ P = 0.19
Marital status	Single	39.76 (4.14)	45.00 (4.43)	68.92 (6.51)
	Married	38.90 (438)	45.61 (3.85)	65.69 (7.32)
	Divorced or widowed	35.83 (4.35)	43.83 (2.78)	65.16 (4.35)
	Test P	F = 1.92^b^ P = 0.15	F = 0.69^b^ P = 0.50	F = 1.22^b^ P = 0.29
Education level	Illiterate	38.79 (4.38)	44.37 (3.48)	66.41 (6.48)
	Primary and secondary schools	38.14 (3.95)	45.11 (3.33)	64.71 (7.52)
	High school and diploma	39.37 (5.01)	47.16 (4.88)	67.66 (6.72)
	Academic	42.33 (3.90)	46.22 (4.40)	69.66 (5.89)
	Test P	F = 2.70^b^ **P = 0.04**	F = 2.56 ^b^ P = 0.058	F = 1.97 ^b^ P = 0.12
Employment status	Unemployed	38.22 (5.45)	44.67 (3.76)	65.58 (1.76)
	Homemaker	39.16 (3.75)	45.61 (3.38)	67.09 (5.54)
	Retired	38.86 (4.00)	46.09 (4.35)	64.88 (6.39)
	Employed	39.33 (4.23)	44.93 (3.55)	67.93 (5.37)
	Test P	F = 0.32 ^b^ P = 0.81	F = 0.91 ^b^ P = 0.43	F = 0.99 ^b^ P = 0.39
Use of assistive devices	Nothing	38.89 (4.46)	45.54 (4.30)	67.54 (6.20)
	Canes and crutches	37.88 (4.05)	45.11 (2.53)	63.03 (5.58)
	Walkers	41.57 (2.99)	45.71 (2.13)	58.14 (13.74)
	Test P	F = 2.04 ^b^ P = 0.13	F = 0.13 ^b^ P = 0.87	F = 9.69 ^b^ **P < 0.001**
Ability to perform daily tasks	Excellent	38.0 (4.30)	43.69 (2.83)	66.38 (3.66)
	Good	38.57 (3.75)	45.88 (4.27)	65.68 (8.49)
	Fair	39.541 (4.35)	45.39 (3.45)	66.34 (5.94)
	Test P	F = 0.75 ^b^ P = 0.47	F = 1.75 ^b^ P = 0.17	F = 0.12 ^b^ P = 0.87
Number of hemodialysis sessions per week	Two times	38.92 (2.85)	46.04 (3.28)	66.84 (7.80)
	Three times	38.81 (4.68)	45.30 (4.01)	65.8 (7.00)
	Test P	t= 0.11^a^ P = 0.91	t = 0.84^a^ P = 0.40	t = 0.64^a^ P = 0.52

^a^ Independent t- test

^b^ ANOVA

#### The association between acceptance of disability and variables of hope and social relational quality

Pearson’s correlation test was used to examine the associations between disability acceptance and the variables of hope and social relational quality. The results displayed in Table 6 revealed a significant, but weak, positive relationship between disability acceptance and its subscales and hope (p < 0.05). There was a significant, but weak, positive relationship existed between disability acceptance and social relational quality (r = 0.18, p = 0.04). The social relational quality showed a significant association with the “subordination of physique” (r = 0.29, p = 0.001), and “transformation from comparative to asset values” (r = 0.22, p = 0.01) subscales of disability acceptance (Table 6).

**Table 6:** Pearson’s correlation between acceptance of disability and it dimensions, and hope and social relational quality in the hemodialysis patients

	Hope	Social relational quality
Acceptance of disability	r = 0.29** **P = 0.001**	r = 0.18* **P = 0.04**
*Dimensions of acceptance of disability*		
Enlargement of scope of values	r = 0.29** **P = 0.001**	r = 0.14 P = 0.12
Subordination of physique	r = 0.23** **P = 0.01**	r = 0.29** **P = 0.001**
Containment of disability effects	r = 0.19* **P = 0.03**	r = 0.09 P = 0.30
Transformation from comparative to asset values	r = 0.21* **P = 0.02**	r = 0.22** **P = 0.01**

* Correlation is significant at the 0.05 level (2-tailed)

** Correlation is significant at the 0.01 level (2-tailed)

#### The prediction of the hemodialysis patients’ acceptance of disability

According to the regression model (acceptance of disability = 41.46 + 0.44 hope + 0.31 social relational quality + 1.24 gender – 0.15 age), a unit improvement in hope was accompanied by a 0.44-unit increase in the disability acceptance score. Moreover, a unit improvement in social relational quality was accompanied by a 0.31-unit increase in the disability acceptance score. This model explained 16% of the disability acceptance (Table 7).

**Table 7:** Multiple linear regression analysis predicting the hemodialysis patients’ acceptance of disability

Model ^a^	Unstandardized coefficients		Standardized coefficients	t	P-value
	B	Std. error	Beta		
1 (constant)	41.46	8.77		4.72	**< 0.001**
Hope	0.44	0.14	0.27	3.17	**0.002**
Social relational quality	0.31	0.15	0.17	1.99	**0.04**
Age	-0.15	0.05	-0.26	-3.12	**0.002**
Gender	1.24	1.30	0.08	0.95	0.34

a. Dependent variable: acceptance of disability; r = 0.43, Unadjusted R square = 0.18, Adjusted R square = 0.16

Age, and gender as confounders

## Discussion

To the best of our knowledge, this is the first study conducted on hemodialysis patients in Iran to evaluate the predictive role of hope and social relational quality on disability acceptance. We collected data from three centers using a systematic sampling approach, which improves the representativeness of the target population and so the generalizability of the findings. Hope and social relational quality predicted disability acceptance.

This study showed that Persian versions of SRQS and ADS were valid and reliable. They had face and content validities. The convergent validity of the construct of both scales was adequate. Similarly, in a study on Chinese cancer patients, the convergent validity of SRQS was assessed and it was reported that SRQS had a significant positive correlation with the optimism scale. Moreover, the good criterion validity of this scale was indicated [31]. In another study, Groomes and Linkowski approved the ADS’ construct validity using factor analysis and the factor loading coefficients ranged from 0.25 to 0.74 [35].

The results of this study showed that Cronbach’s alpha of SRQS and ADS was 077, and 0.80, respectively; therefore, both scales’ internal consistency is supported. In a study regarding the psychometrics properties of SRQS, it was reported that Cronbach’s alpha of SRQS was 0.83 [26]. In a previous study carried out by Chiang et al. on chronic kidney disease patients, the reliability of ADS was confirmed by Cronbach’s alpha of 0.92 [21].

This study showed hope in hemodialysis patients predicted disability acceptance. This finding is consistent with other studies that indicated that disability acceptance was correlated to hope in patients with chronic conditions such as laryngectomy [38], burn [25], and traumatic paraplegia [39]. Moreover, disability acceptance predicted hope in adults with physical disabilities [40]. Patients’ hope improved their acceptance and coping [41]. Hope could also support the role of disability identity in promoting well-being (e.g., agency and pathways) among adults with physical disabilities [40].

This study also indicated that social relational quality predicted disability acceptance. Zhang et al. reported a significant relationship between the disability acceptance score and the total score of social relational quality and its subscales such as family commitment and friendship [26]. Disability acceptance may improve self-esteem [42] and quality of life dimensions such as social relationships [43]. In other words, social support and disability acceptance improve patients’ quality of life [44].

This study indicated the mean score of disability acceptance was significantly higher in the people who did not use any aids to move compared to others. In fact, hemodialysis patients who had to use canes, crutches, and walkers reported lower mean score of disability acceptance. In a study on a chronic disease such as multiple sclerosis, it was reported that patients with physical disability showed lower mean scores of illness’ acceptance and quality of life [45].

The current study showed hemodialysis patients with academic degree reported highest mean score of hope. In other words, patients with higher level of education reported higher mean score of hope. Similarly, Yaghoobzadeh et al. revealed that patients with chronic diseases such as cardiovascular diseases who had higher education level indicated higher level of hope [46]. In another study on women after cardiac surgery, it was indicated patients with lower education had lower level of hope [47].

One of the strengths of this study was that psychosocial concepts such as hope, disability acceptance, and social relational quality using valid scales. To improve evidence-based practice, assessment of ESRD patients’ hope, disability acceptance, and social relational quality are recommended. Moreover, medical sciences students, such as medicine, nursing, rehabilitation etc., should be provided with information regarding these psychosocial concepts in their classrooms.

Moreover, this cross-sectional and predictive study contributes to understanding the relationships between different variables. Therefore, healthcare workers and their managers are recommended to consider strategies to improve the hope and quality of social relationships in hemodialysis patients and probably their disability acceptance. In addition, the validity and reliability of Persian versions of SRQS and ADS were analyzed and approved in hemodialysis patients. Therefore, it is recommended that evidence-based research should be improved through using these valid and reliable scales in hemodialysis patients.

A cross-sectional design, which did not confirm the cause-effect relationship, was a limitation of this study. Further studies are recommended to be conducted on larger sample sizes in different parts of the world to improve the generalizability of the findings.

Given the association between disability acceptance, and hope and social relational quality, it is suggested that the hope level, as well as the social-relational quality in the individuals undergoing hemodialysis, should be improved in clinical settings, thereby helping them accept their disabilities.

## Conclusion

The study showed that disability acceptance was associated with higher hope and social relational quality levels. Interventions development is recommended to improve hope and social relational quality in patients undergoing hemodialysis to enhance their disability acceptance.

## Data Availability

The datasets used and/or analyzed during the current study are available from the corresponding author on reasonable request.
